# Tetrahedral DNA nanostructure improves transport efficiency and anti‐fungal effect of histatin 5 against *Candida albicans*

**DOI:** 10.1111/cpr.13020

**Published:** 2021-03-11

**Authors:** Bowen Zhang, Xin Qin, Mi Zhou, Taoran Tian, Yue Sun, Songhang Li, Dexuan Xiao, Xiaoxiao Cai

**Affiliations:** ^1^ State Key Laboratory of Oral Diseases National Clinical Research Center for Oral Diseases West China Hospital of Stomatology Sichuan University Chengdu China

**Keywords:** anti‐microbial peptides, *Candida albicans*, Fungi, Histatin 5, Tetrahedral framework nucleic acids

## Abstract

**Objectives:**

Anti‐microbial peptides (AMPs) have been comprehensively investigated as a novel alternative to traditional antibiotics against microorganisms. Meanwhile, Tetrahedral DNA nanostructures (TDNs) have gained attention in the field of biomedicine for their premium biological effects and transportation efficiency as delivery vehicles. Hence, in this study, TDN/Histatin 5 (His‐5) was synthesized and the transport efficiency and anti‐fungal effect were measured to evaluate the promotion of His‐5 modified by TDNs.

**Materials and Methods:**

Tetrahedral DNA nanostructures/His‐5 complex was prepared via electrostatic attraction and characterized by transmission electron microscopy (TEM), polyacrylamide gel electrophoresis (PAGE), dynamic light scattering (DLS) and electrophoretic light scattering (ELS). The anti‐fungal effect of the TDN/His‐5 complex was evaluated by determining the growth curve and colony‐forming units of *C. albicans*. The morphological transformation of *C. albicans* was observed by light microscope and scanning electron microscope (SEM). Immunofluorescence was performed, and potassium efflux was detected to mechanistically demonstrate the efficacy of TDN/His‐5.

**Results:**

The results showed that Histatin 5 modified by TDNs had preferable stability in serum and was effectively transported into *C. albicans*, leading to the increased formation of intracellular reactive oxygen species, higher potassium efflux and enhanced anti‐fungal effect against *C. albicans*.

**Conclusions:**

Our study showed that TDN/His‐5 was synthesized successfully. And by the modification of TDNs, His‐5 showed increased transport efficiency and improved anti‐fungal effect.

## Graphical abstract

TDN/His‐5 were assembled by TDNs and Histatin 5 by electronic attraction and internalized by Candida albicans, leading to the increased formation of intracellular reactive oxygen species, high potassium efflux, and enhanced antifungal effect against C. albicans.

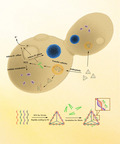

## 1 INTRODUCTION

Nowadays, diseases caused by eukaryotic microorganisms have become global contributors to illness and death, and this threat is intensely debated.^1^, ^2^, ^3^, ^4^ Among the large variety of eukaryotes, fungi and especially *Candida albicans*, are the predominant causative agents of invasive infections. They can affect different tissues and systems, ranging from the mucosa to the haematological system.^5^, ^6^ Despite the advances and availability of an emerging and more extensive therapeutic arsenal, current progress in the control of fungal diseases remains limited because of the gradual development of drug resistance in fungi, which urges aggressive and immediate action for developing novel methods.^4^, ^7^, ^8^

Positively charged short anti‐microbial peptides (AMPs) found in plants and animals are critical components of the innate immune system and defend against invading microorganisms.^9^ Due to its broad‐spectrum anti‐biofilm activity and ability to modulate the host immune response, AMPs showed clear advantages over conventional anti‐microbials.^10^ Moreover, AMPs showed distinctive anti‐microbial activities like formation of ion channels, transmembrane pores and extensive membrane rupture for extracellular AMPs, and inducing loss of ATP, misfolded proteins and aseptate filaments for intracellular AMPs. Therefore, AMPs presented the great value for conventional drug resistance.^11^, ^12^

As for extracellular AMPs, the initial stage of their function depends on the passive combination between their positively charged domain and the negatively charged microbial membrane.^13^, ^14^ Therefore, the modification of extracellular AMPs is mainly focused on enhancing its electrostatic property and strengthening the electrostatic and hydrophobic attraction between AMPs and cell membrane including amending the peptide sequence, altering the nature and position of the organometallic group and assembling the characteristic domain.^15^, ^16^, ^17^ However, it is more important for intracellular AMPs to be internalized by cells for their intracellular target mechanisms. Moreover, due to their various cellular uptake pathways, like lipid‐raft‐dependent macropinocytosis for TAT‐fusion proteins, permease‐mediated translocation for apidaecin and receptor‐mediated mechanism for histatin, the delivery carrier that can be internalized by diverse cells actively and loaded with different drugs is required for the modification and improvement for intracellular AMPs.^18^, ^19^, ^20^

What is more, distinct from the bacterial cell wall, the fungal cell wall is composed of mannoproteins, chitins and α‐,β‐glucans, which weakens the initial binding of fungal cell membrane with AMPs, leading to decreased AMPs delivery efficiency and anti‐fungal effect.^17^, ^21^ Therefore, for the anti‐fungal AMPs like His‐5, a novel way to optimize them is needed to overcome the obstacles associated with the special cell well structure of fungi.

Recently, tetrahedral DNA nanostructures (TDNs) have attracted increasing interest because of their editability and biocompatibility.^22^, ^23^, ^24^, ^25^ Despite the polyanionic nature of DNA, TDNs can be internalized via endocytosis and transported into lysosomes in a microtubule‐dependent manner.^26^, ^27^ Accordingly, TDN‐based drug delivery has been proposed and researched extensively at the cellular and bacterial levels over the past decade.^24^, ^28^, ^29^ From previous studies, TDNs have yielded satisfactory results as delivery vehicles attached with low‐molecular‐weight drugs like nucleic acids, antibiotics and peptides.^28^, ^30^, ^31^ This implied that TDNs can be used to load intracellular anti‐fungal AMPs like His‐5 to improve their transport efficiency and anti‐fungal ability.

In this study, Histatin 5 was selected as the representative of intracellular anti‐fungal AMPs and combined with TDNs to construct TDN/His‐5 complex, whose characteristics and anti‐fungal effects were tested. To our knowledge, this is the first study to design a combination of TDNs and intracellular AMP as anti‐fungal agents. Owing to electrostatic attraction, the cationic AMP His‐5 can bind easily to anionic TDNs, leading to more efficient uptake by *C. albicans*.^32^, ^33^ This approach provides a novel alternative for further modification of peptide drugs and exhibits the versatile potential of TDNs in anti‐fungal drug delivery.

## 2 MATERIALS AND METHODS

### 2.1 Cell culture

*Candida albicans* SC5314 (wild type) obtained from our laboratory were commercially from the American Type Culture Collection.^34^ *Candida albicans* strains were propagated on liquid or solid YPD medium at 30°C. The yeast cells used in all experiments were in the exponential growth phase (OD_600_ = 0.6). For inducing hyphae, *C. albicans* cells were grown in RPMI 1640 medium containing 2.5% foetal calf serum, 2 mm L‐glutamine, 20 mm HEPES and 16 mm sodium hydrogen carbonate (pH 7.0) (RP medium) at 37°C.

### 2.2 Tetrahedral DNA nanostructures preparation and verification

Tetrahedral DNA nanostructuress were synthesized by four single‐stranded DNAs (deoxyribonucleic acids) (Table 1) purchased from Takara Bio (Otsu, Japan).^23^ Equimolar concentrations of S1, S2, S3 and S4 were mixed in Tris‐maleate (TM) buffer (50 mm MgCl_2_.6H_2_O, 10 mm Tris‐HCl). The total mixture was placed into a PCR instrument, and heated to 95°C for 10 minutes then cooled rapidly to 4°C for 20 minutes.^35^, ^36^ The primary TDNs were purified by ultrafiltration (Amicon Ultra 10K device) with phosphate‐buffered saline (PBS). Transmission electron microscopy (TEM) was applied to analyse the morphology of the TDNs and 8% PAGE was applied to characterize single‐stranded DNAs and TDNs.

**TABLE 1:** Base Sequence of Single‐Strand DNA

ssDNA	Base sequence
Cy5‐Strand1	5'‐Cy5‐ATTTATCACCCGCCATAGTAGACGTATCACCAGGCAGTTGAGACGAACATTCCTAAGTCTGAA‐3'
Strand 1	5'‐ATTTATCACCCGCCATAGTAGACGTATCACCAGGCAGTTGAGACGAACATTCCTAAGTCTGAA‐3'
Strand 2	5'‐ACATGCGAGGGTCCAATACCGACGATTACAGCTTGCTACACGATTCAGACTTAGGAATGTTCG‐3'
Strand 3	5'‐ACTACTATGGCGGGTGATAAAACGTGTAGCAAGCTGTAATCGACGGGAAGAGCATGCCCATCC‐3'
Strand 4	5'‐ACGGTATTGGACCCTCGCATGACTCAACTGCCTGGTGATACGAGGATGGGCATGCTCTTCCCG‐3'

### 2.3 Synthesis and characterization of the TDN/His‐5 Complex

Histatin 5 (Bankpeptide Hefei Biotechnology Co., Ltd.) dissolved in doubled distilled H_2_O (ddH_2_O) and TDNs were mixed in different ratios (His‐5: TDNs = 800:1, 400:1, 200:1, 100:1, 50:1, 25:1) and incubated for 30 minutes at room temperature.^37^ Then, the mixtures were ultrafiltered (Amicon Ultra 10K device) to eliminate the unloaded compound and diluted in the PBS. To verify the successful assembly and detect the optimum mixture ratio, 8% PAGE was used to determine the molecular weights of the TDNs and TDN/His‐5 complexes, which were mixed in different ratios as mentioned above. The zeta potential and the hydrodynamic size of the TDN/His‐5 were measured to confirm the PAGE results.^38^

### 2.4 Serum stability test of TDN/His‐5 complex

To verify stability of TDN/His‐5 complex in the serum medium, matrix‐assisted laser desorption ionization time of flight mass spectrometry (MALDI‐TOF MS) was utilized to assess the degradation profile of His‐5. His‐5 and TDN/His‐5 incubated in the 10% foetal calf serum medium for 6h were measured by MALDI‐TOF MS. And the proportions of peak areas (mass range from 2900 to 3000) to the total area in spectrum were calculated as the proportions of His‐5 in the medium. The mass percentage of His‐5 was calculated with following equation*:*

$$\text{Mass}\;\text{Percentage}\;\text{of}\;\text{Histatin}\;5\;\left(\%\right)=\frac{\text{Peak}\;\text{area}\;\text{of}\;\text{Histatin}\;5}{\text{Total}\;\text{area}\;\text{of}\;\text{sample}}\times 100\%\;$$

Initial His‐5 and TDN/His‐5 in the 10% foetal calf serum medium were measured as control groups. And the proportions were compared between the TDN/His‐5 groups and His‐5 groups.

### 2.5 Loading efficiency analysis

The loading efficiency of TDN/His‐5 complex was determined using ultrafiltration and spectrophotometry. TDNs and His‐5 were incubated for 30 minutes at room temperature in different ratios (1:50, 1:100, 1:200). Then, the TDN/His‐5 solutions were ultrafiltered (Amicon Ultra 10K device, USA) at 9 600 *g* for 10 minutes, and the concentrations of unloaded His‐5 in the remaining solutions were measured and calculated by spectrophotometry at the wavelength of 214 nm. The loading efficiency of His‐5 onto TDNs was calculated with the following equation:

$$\Delta =\frac{C_{1}‐C_{2}}{C_{2}}\times 100\%$$

*C*_1_ and *C*_2_ mean the initial and unloaded concentration of Histatin 5, respectively.

### 2.6 Growth assay

To evaluate the yeast killing ability of the TDN/His‐5 complex, exponential‐phase *C. albicans* was used to perform fungicidal activity assays. *C. albicans* cells were washed with PBS thrice and diluted to 10^6^ CFU/mL. Then, the strains were seeded at an initial density of 5 × 10^5^ CFU/mL, divided into three groups and treated with 400 nm TDNs, 20 µm Histatin 5, TDN/His‐5 complex (400 nm TDNs with 20 µm His‐5) for 1:50 mass ratio group; 200 nm TDNs, 20 µm Histatin 5, TDN/His‐5 complex (200 nm TDNs with 20 µm His‐5) for 1:100 mass ratio group, 100 nm TDNs, 20 µm Histatin 5, TDN/His‐5 complex (100 nm TDNs with 20 µm His‐5) for 1:200 mass ratio group, respectively, in a 96‐well plate in YPD liquid medium. In addition, YPD liquid medium seeded with *C. albicans* alone was used as a positive control, in contrast, non‐seeded YPD liquid medium with the TDN/His‐5 complex was used as a negative control. Fungal growth was measured with an automated spectrophotometer at 30°C, and the optical density at 600 nm was detected automatically over 24 hours. The OD_600_ value was detected once an hour, and the plate was shaken every 15 minutes to avoid precipitation.^39^, ^40^ Finally, the anti‐fungal rate was calculated with the following equation:

$$\text{Anti ‐ fungal}\;\text{Rate}\;\left(\text{\textbackslash{}\%}\;\right)=\frac{\text{Positive}\;\;\text{control}\;\;{\text{OD}}_{600}‐\text{Experimental}\;\;\text{group}\;{\text{OD}}_{600}}{\text{Positive}\;\;\text{control}\;{\text{OD}}_{600}‐\text{Negative}\;\;\text{control}\;\;{\text{OD}}_{600}}\times 100\%$$

Furthermore, *C. albicans* were treated with 200 nm TDNs, 20 µm Histatin 5 or TDN/His‐5 complex of three mass ratio (400 nm TDNs, 20 µm His‐5; 200 nm TDNs, 20 µm His‐5 and 100 nm TDNs, 20 µm His‐5) in 96‐well plates as an density of 5 x 10^5^ CFU/mL for 2 hours at 30°C in YPD liquid medium.^41^ Then, the cells were rinsed with PBS thrice and diluted with YPD liquid medium. Fungal medium without any interference was used as a positive control. Five hundred cells from each well were plated on YPD agar medium and incubated for 24 hours at 30°C. Finally, the number of colony‐forming units (CFUs) was counted andanalysed.^42^

### 2.7 Morphology assay

To determine the morphology of *C. albicans* under the TDN/His‐5 complex conditions, the yeast cells were diluted to a concentration of 1 x 10^6^ CFU/mL in a 12‐well plate in RP medium and treated with 200 nm TDNs, 20 µm Histatin 5 or 200 nm TDN/His‐5 complex (1:100 mass ratio) for 8 hours at 37°C.^43^ After that, the samples were rinsed thrice with PBS and fixed with 4% paraformaldehyde overnight at 4°C. After fixation, the samples were dehydrated with a gradient series of ethanol. Specimens were observed under a Leica DM1000 microscope (Leica Microsystems) and a scanning electron microscope (Inspect F, FEI, America).^40^, ^44^

### 2.8 Qualitative analysis of TDN/His‐5 uptake by *Candida albicans*

Cyanine‐5 (Cy4)‐labelled TDNs and 6‐carboxyfluorescein (FAM)‐labelled Histatin 5 were applied for the observation of cellular uptake of TDN/His‐5 by confocal laser scanning microscopy (CLSM). *C. albicans* were cultured in RP medium in 96‐well plates and diluted to a density of 10^6^ CFU/mL. In addition, 200 nm Cy5‐TDNs, 20 µm FAM‐Histatin 5 and 200 nm Cy5‐TDN/FAM‐Histatin 5 (1:100 mass ratio) were added to different wells for 2 hours at 37°C. After the incubation, the cells were rinsed with PBS three times and stained with Hoechst for 30 minutes. Then, the suspension was rewashed and resuspended in PBS into a density of 107 CFU/mL. Five microliters of the samples was added to a slide and covered with a coverslip, which was observed with a CLSM (N‐SIM, Nikon; Excitation wavelength: 492 nm and Emission wavelength: 518 nm for FAM; Excitation wavelength: 650 nm and Emission wavelength: 670 nm for Cy5).^45^ The fluorescence intensities of confocal images were analysed by Image J.

### 2.9 Quantitative analysis of TDN/His‐5 uptake by *Candida albicans*

Exponential‐phase yeast cells (1 × 10^6^ CFU/mL) were treated with FAM‐Histatin 5 (20 µm) and TDN/FAM‐His‐5 (200 nm, 1:100 mass ratio) 3 or 6 hours at 30°C. Then, the samples were harvested and rinsed with PBS three times. The samples were resuspended in PBS at a concentration of 1 x 10^6^ CFU/mL in flow tubes. And the untreated samples were used as the negative control group. All the samples were detected by flow cytometry (FC500; Beckman) (Excitation wavelength: 492 nm and Emission wavelength: 518 nm for FAM).^33^, ^46^

### 2.10 Measurement of intracellular ROS levels

Both yeast‐form and hyphae‐form *C. albicans* cells were cultured at a density of 1 × 10^6^ CFUs/mL. The strains were incubated with TDNs (200 nm), Histatin 5 (20 µm) and TDN/His‐5 (200 nm, 1:100 mass ratio) for 3 hours. After that, the samples were resuspended with PBS three times (1500 *g*, 5 minutes), incubated with DCFH‐DA solution (1:1000) for 25 minutes and resuspended again. Then, the samples were stained with Hoechst for 30 minutes, rinsed with PBS three times and diluted into a density of 1 x 10^7^ CFU/mL. Five microliters from each sample was added to a slide and covered with a coverslip, which was observed with a CLSM (Excitation wavelength: 350 nm and Emission wavelength: 460 nm for Hoechst, Excitation wavelength: 492 nm and Emission wavelength: 518 nm for DCF). The immunofluorescence images were analysed by Image J.

Moreover, after incubation with DCFH‐DA solution as mentioned above, parts of yeast‐form samples were rinsed thrice with PBS (4000 rpm, 5 minutes) and diluted into a density of 1 x 10^6^ CFU/mL. 1 × 10^4^ yeast cells were selected and analysed using a flow cytometer to deter the mean fluorescence intensity of DCF (Excitation wavelength: 492 nm, Emission wavelength: 518 nm for DCF).^47^, ^48^

### 2.11 Determination of potassium efflux from *Candida albicans*

Exponential‐phase yeast cells were rinsed with PBS thrice (4000 rpm, 5 minutes) and diluted into a density of 1 × 10^6^ CFU/mL in YPD liquid medium. Then 200 µL from each sample was incubated with TDNs (200 nm), Histatin 5 (20 µm) and TDN/His‐5 (200 nm, 1:100 mass ratio) at 30°C for 4 hours. After that, the tubes were centrifuged, and the supernatants were collected and diluted 1:2 with ddH_2_O for testing. The supernatants were quantitated for their potassium concentration by atomic absorption spectroscopy (AAS; SpectrAA 220FS, VARIAN).

### 2.12 Statistical analysis

All the results presented are based on at least three individual experiments. Statistical analyses were executed in GraphPad Prism (GraphPad Inc). Student's *t* test was used, and the significance level was set to α = 0.05.

## 3 RESULTS AND DISCUSSION

In this study, we selected His‐5 as the representative of intracellular anti‐fungal AMPs and combined it with TDNs to construct the TDN/His‐5 complex. By the interaction between TDNs and the fungal cell membrane, cellular uptake and anti‐fungal efficacy of His‐5 were improved.

### 3.1 Synthesis and characterization of TDNs and the TDN/Histatin 5 complex

**FIGURE 1:**
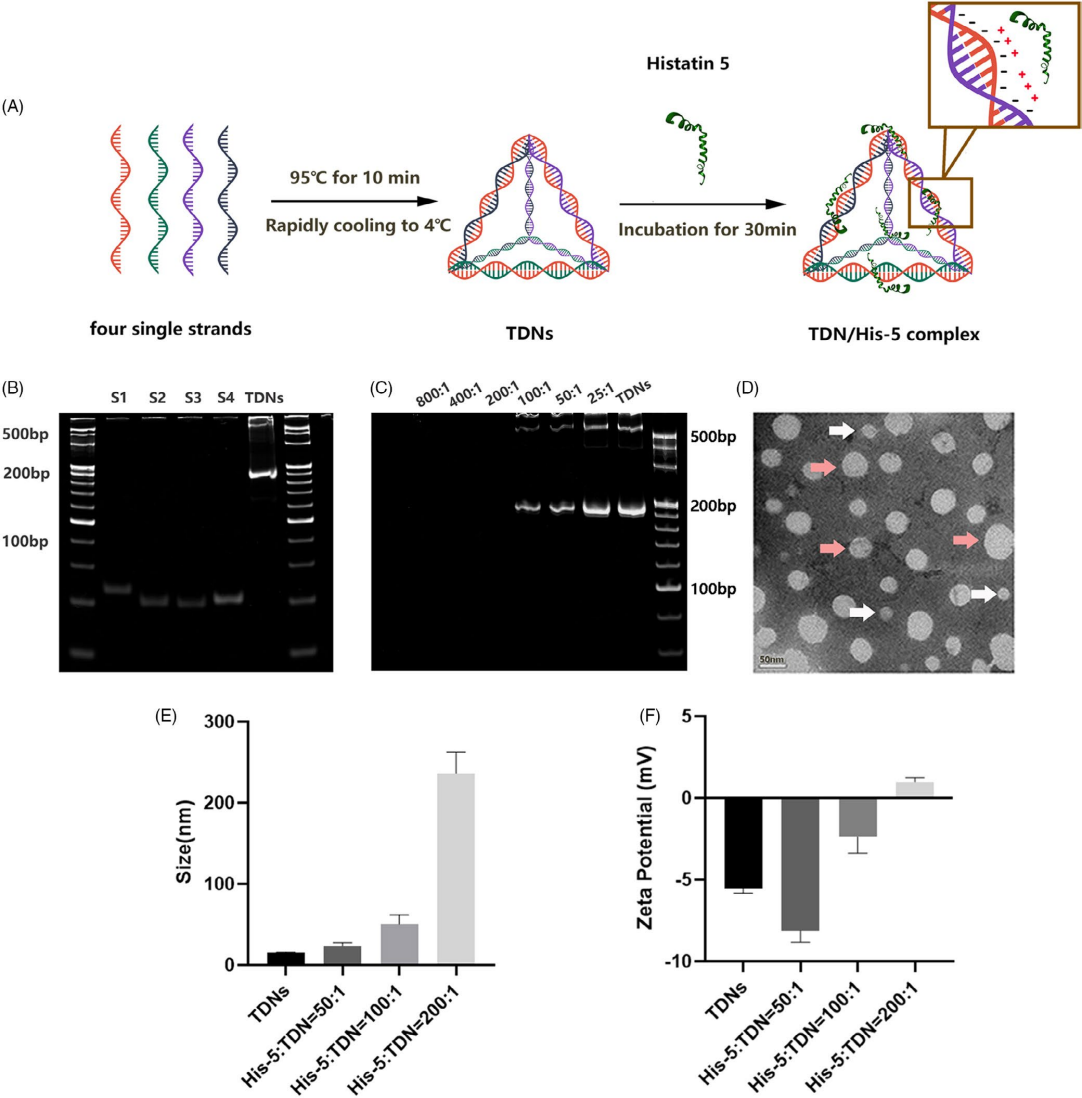
Characterization of TDN/His‐5 complex. (A) The scheme of synthesis and delivery of TDN/His‐5 complex. (B) Analysis of the relative size of single‐stranded DNA (S1, S2, S3 and S4) and TDNs. (C) Analysis of the electrophoretic migration of the TDN/His‐5 complex at different gradient mixture ratios (His‐5: TDN = 800:1, 400:1, 200:1, 100:1, 50:1, 25:1) and TDNs. (D) Transmission electron microscope image of the synthesized TDNs. White arrow points to successfully synthesized TDNs. Pink arrow points to polymers. (E) Analysis of the hydrodynamic sizes of TDNs and TDN/His‐5 complexes (His‐5/TDN = 200:1, 100:1, 50:1). (F) Analysis of the zeta potentials of TDNs and TDN/His‐5 complexes (His‐5: TDN = 200:1, 100:1, 50:1)

Conventionally, TDNs comprise four different single‐stranded DNA (ssDNA) and self‐assemble according to the specific sequence of ssDNA based on complementary pairing rules (Figure 1A).^25^ Eight percent PAGE was performed to evaluate the synthesis and characterization of TDNs. Figure 1B showed the successful synthesis of TDNs ranging around 200 bp in size. The morphology of TDNs was observed using transmission electron microscopy, and we found that TDNs comprise some monomers and polymers (Figure 1D). To verify the optimal mixture ratio, TDNs were combined with a gradient concentration of His‐5, and the mixtures were characterized by 8% PAGE. As demonstrated in Figure 1C, at peptide: TDN mass ratios of 25:1, 50:1 and 100:1, the TDN/His‐5 complex migrated to non‐complexed TDNs. In contrast, the electrophoretic mobility of the complex was completely inhibited at the ratio of 200:1, indicating that the His‐5 was sufficient to neutralize the charge of TDNs at the ratio of 200:1.

Furthermore, the zeta potentials and hydrodynamic sizes were measured to prevent excessive agglomeration of TDN/His‐5 complex owing to an imbalance of the mixture ratio, which may affect the transport efficiency. The average zeta potential of TDN/His‐5 complex at the peptide/TDN ratios of 50:1 and 100:1 was −8.13 and −2.37, respectively, whereas that at the ratio of 200:1 was reversed to positive charge (0.98; 95% CI: 0.39‐1.57) (Figure 1F) (Table 2). The hydrodynamic size of the complex increased significantly with increasing concentration ratio. This size at a peptide/TDN ratio of 200:1 exceeded 200 nm, which has been previously reported to weaken the internalization of the complex (Figure 1E) (Table 2).^26^, ^49^

**TABLE 2:** Statistics of the hydrodynamic sizes and zeta potentials of the TDN/His‐5 complexes (His‐5: TDN = 200:1, 100:1, 50:1)

	Size (nm)	Zeta potential (mV)
TDNs	11.32, 95%CI: [11.12, 12.47]	−5.51, 95%CI: [−6.20, −4.82]
His‐5: TDNs = 50:1	21.55, 95%CI: [17.29, 24.70]	−8.13, 95%CI: [−9.71, −6.55]
His‐5: TDNs = 100:1	47.78, 95%CI: [40.91, 59.12]	−2.37, 95%CI: [−4.66, −0.08]
His‐5: TDNs = 200:1	221.73, 95%CI: [192.27, 257.93]	0.98, 95%CI: [10.39, 1.57]

### 3.2 Serum stability and loading efficiency test of TDN/His‐5 complex

**FIGURE 2:**
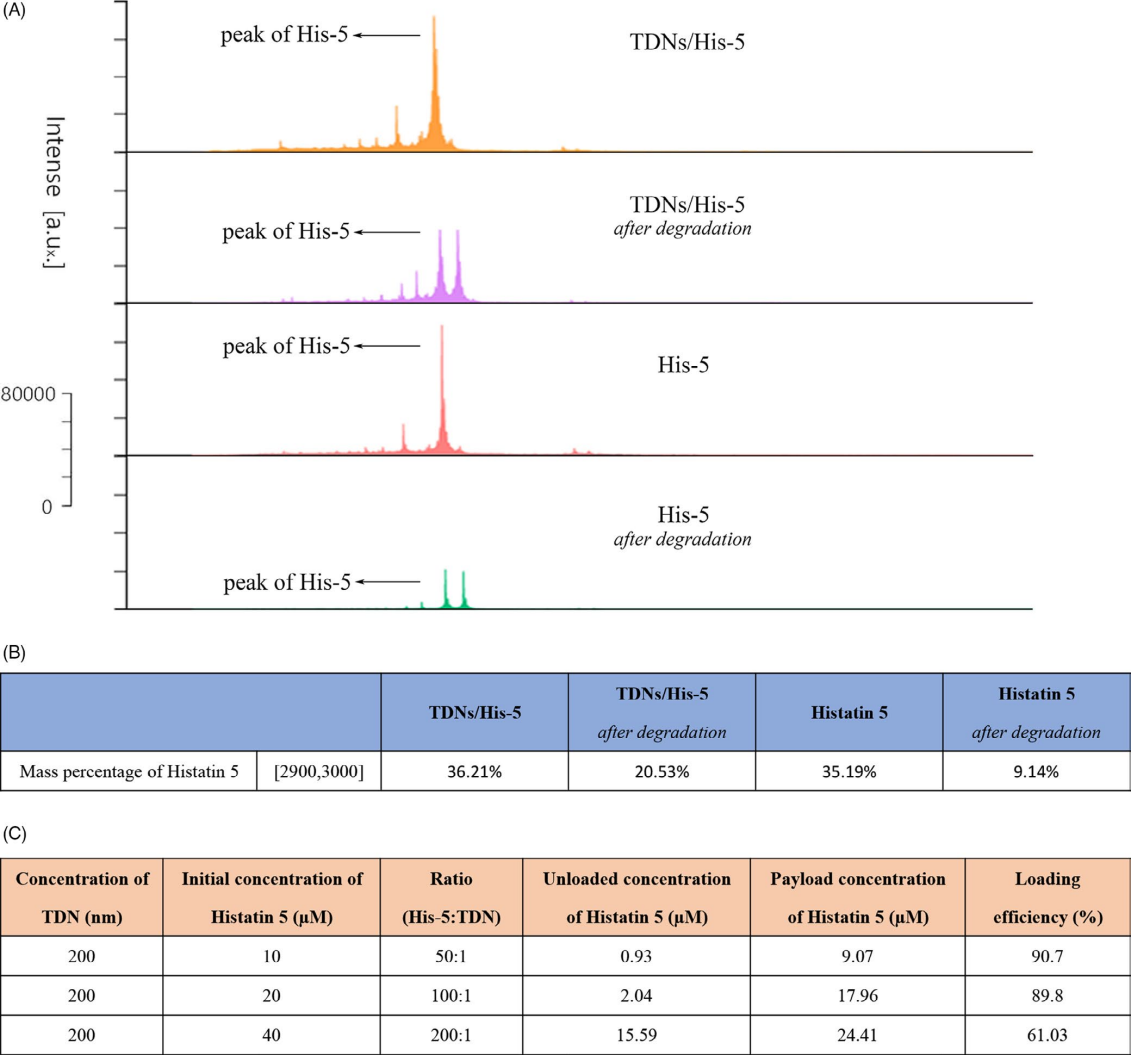
Stability analysis of His‐5 and TDN/His‐5 complex. (A) Degradation profiles of His‐5 and TDN/His‐5 in the 10% foetal calf serum medium measured by MALDI‐TOF MS, within the *m/z* range of 2900‐3000. (B) Corresponding mass percentage of His‐5. (C) Analysis of the payload rate of the TDN/His‐5 complexes (His‐5: TDN = 200:1, 100:1, 50:1)

An obvious difficulty in the application of AMP is its vulnerability to degradation by serum proteases.^29^, ^50^, ^51^ Previous studies showed that TDNs protected the ‘cargo’ they deliver.^49^, ^52^, ^53^ For further proving the efficacy of the TDN/His‐5 complex, the stability of His‐5 protected by TDNs in a medium containing serum was measured. Firstly, the matrix (the 10% foetal calf serum medium) was detected by MALDI‐TOF MS and the results showed that there was no specific peak within the *m/z* range of 2900‐3000, indicating no interference with His‐5 (Figure S1). Moreover, the peak of His‐5 of TDN/His‐5 complex after incubation was obviously higher than that of intact His‐5 after incubation, within the *m/z* range of 2900‐3000. And the decline of mass percentage of His‐5 after degradation in TDNs/His‐5 group (from 36.21% to 20.53%) was much smaller than that in Histatin 5 group (from 35.19% to 9.0.14%), that indicating more stable ability for His‐5 with the assistant of TDNs. (Figure 2A,B). Moreover, at a peptide/TDN ratio of 200:1, the complex showed a significant decrease in payload rate compared with that at 100:1 and 50:1 ratios, indicating that the binding capacity of His‐5 and TDN decreased at the ratio of 200:1 (Figure 2C). Consequently, for achieving both stable combination and optimal anti‐fungal effect, 100:1 was selected as the optimal ratio of the TDN/His‐5 complex for subsequent experiments.

### 3.3 Anti‐fungal activity assays of the TDN/Histatin 5 complex against *Candida albicans*

**FIGURE 3:**
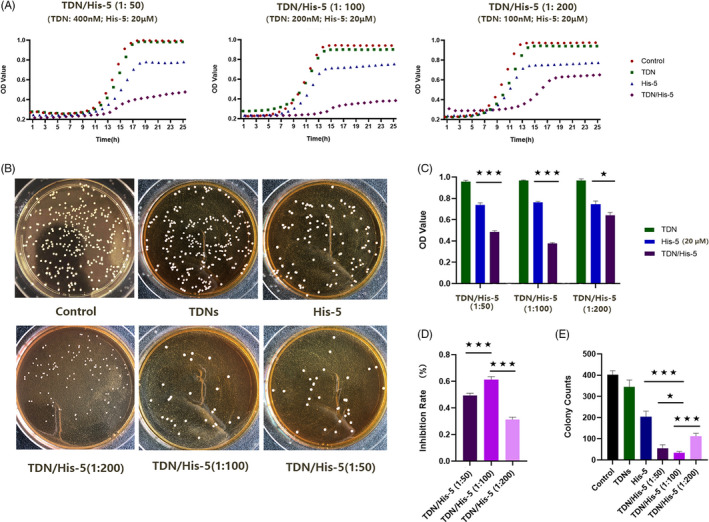
Evaluation of the ability of TDN/His‐5 complex to inhibit fungal growth in *C albicans*. (A) The growth curves of *Candida albicans* treated with TDNs, His‐5 and TDN/His‐5 complex. (B) Images of plate colony count of fungal suspension incubated with TDNs (200 nm), His‐5 (20 µm), TDN/His‐5 (1:200) (TDN: 100 nm; His‐5:20 µm), TDN/His‐5 (1:100) (TDN: 200 nm; His‐5:20 µm), TDN/His‐5 (1:50) (TDN: 400 nm; His‐5:20 µm). (C) Analysis of the final OD value at 24 h of growth. (D) The anti‐fungal efficacy of TDN/His‐5 complex at different mass ratios based on growth curve. (E) Analysis of the colony counts of *C albicans* treated with 200 nm TDNs, 20 µm His‐5 and TDN/His‐5 complex of different mass ratios. Data are represented as means ± standard deviations, n = 3; statistical significance: *P < .05, **P < .01, ***P < .001

For detecting the anti‐fungal activity of drugs against yeast‐form *C. albicans*, the growth curves with TDNs, His‐5 and TDN/His‐5 complex were measured over 24 hours. As shown in Figure 3A,C, in the groups with different TDN/His‐5 mass ratios, the TDN/His‐5 complex presented a more significant anti‐fungal effect than TDNs and His‐5 individually, whereas TDNs did not show obvious effect on the growth of *C. albicans*. Fungal growth was markedly inhibited in the log phase in the TDN/His‐5 complex group compared with other groups. In addition, the anti‐fungal rates of TDN/His‐5 complex groups were calculated based on the growth curves (Figure 3D). TDN/His‐5 of 1:100 mass ratio showed better anti‐fungal effect than 1:50 and 1:200 mass ratio, as evidenced from the results of payload rate experiment and stability experiment. However, the anti‐fungal efficiency of TDN: His‐5 at a mass ratio of 1:50 (when more TDNs combined with His‐5) decreased instead. This may be because of the stronger negative charge of the TDN/His‐5 complex with excessive TDNs, which hinders the contact between the complex and fungal wall and consequently reduces the cellular uptake of the complex.^32^, ^54^ Moreover, the similar results were also showed in the CFUs assay. The number of fungal colonies was the lowest in the group treated with TDN/His‐5 complex (1:100) (34 ± 12). By contrast, in the groups treated with TDN/His‐5 at the mass ratio of 1:50 and 1:200, the CFUs were 54 ± 19 and 112 ± 26, respectively (Figure 3B,E).

**FIGURE 4:**
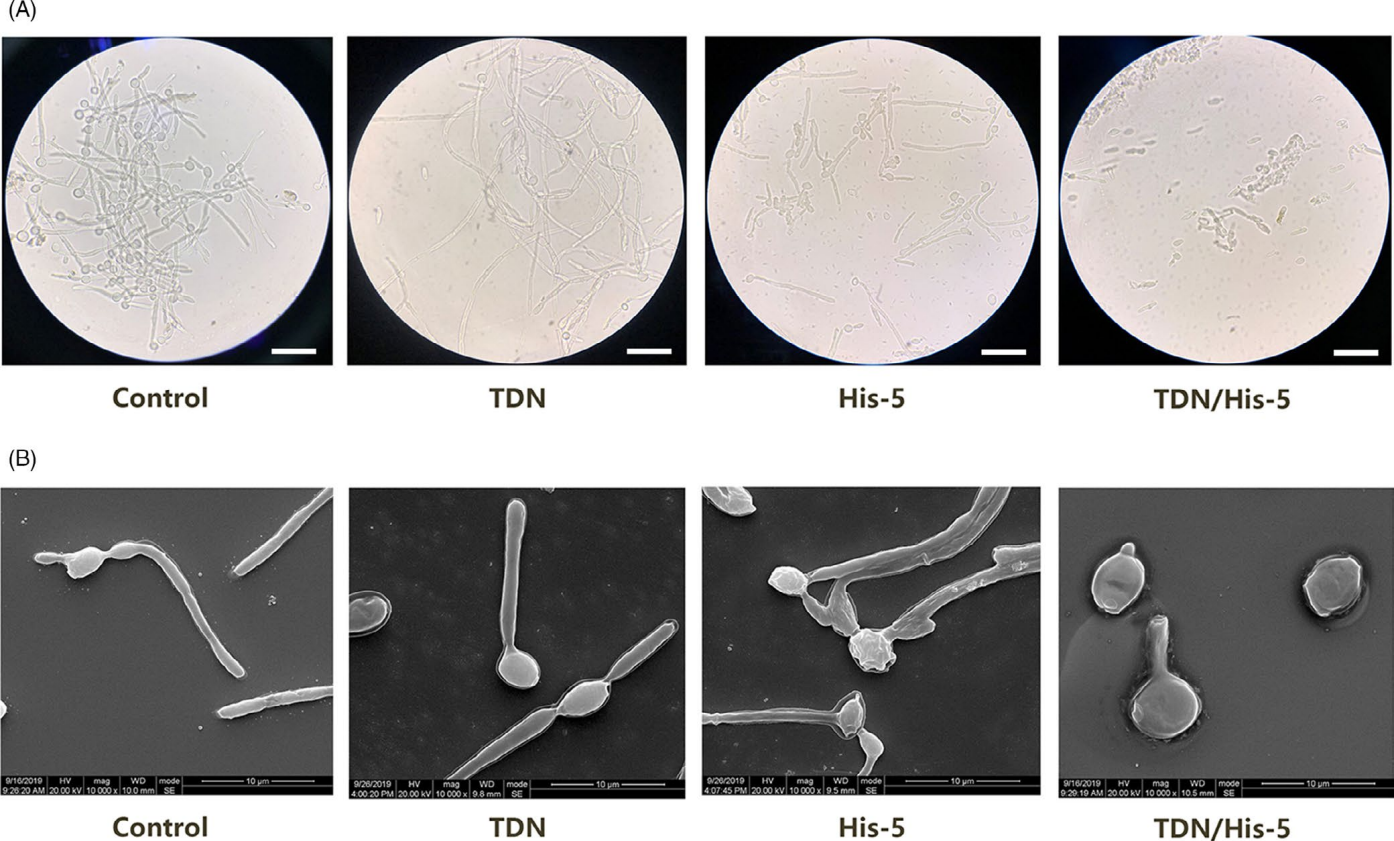
Evaluation of the ability of TDN/His‐5 complex to inhibit the morphological transformation of *Candida albicans*. (A) Bright field image of *C. albicans* treated with 200 nm TDNs, 20 µm His‐5 and 200 nm TDN/His‐5 complex (1:100) in RP medium for 8 h. Scale Bar: 50 µm (B) Observation of *C albicans* treated with 200 nm TDNs, 20 µm His‐5 and 200 nm TDN/His‐5 complex (1:100) in RP medium for 8 h

In the morphology assay, bright field images of the control and TDN groups revealed large amounts of hyphae after culturing in the RP medium (Figure 4A). By contrast, more yeast cells and fewer hyphae were presented in the samples treated with His‐5; the yeast bodies shrank, and the hyphae branches frequently formed bundles. When exposed to the TDN/His‐5 complex, only a few hyphae and yeast cells were detected, indicating that TDN/His‐5 inhibited the growth of the hyphal form of *C. albicans*. Moreover, the image of TDN/His‐5 group showed yeast cell debris indicating the fungicidal effect of TDN/His‐5 complex. Scanning electron microscopy images showed the detailed morphology of *C. albicans* (Figure 4B). Compared with the regular hyphal formation of *C. albicans* in control and TDN groups, the microscopy images of the His‐5 group showed lysis of *C. albicans* membrane during hyphal formation. In the TDNs/His‐5 group, the swelling yeast cells demonstrated efficient inhibition of morphological transformation from the yeast‐form to the hyphal form of *C. albicans* and lysis of yeast cells.

### 3.4 Cellular uptake of the TDN/Histatin 5 complex

**FIGURE 5:**
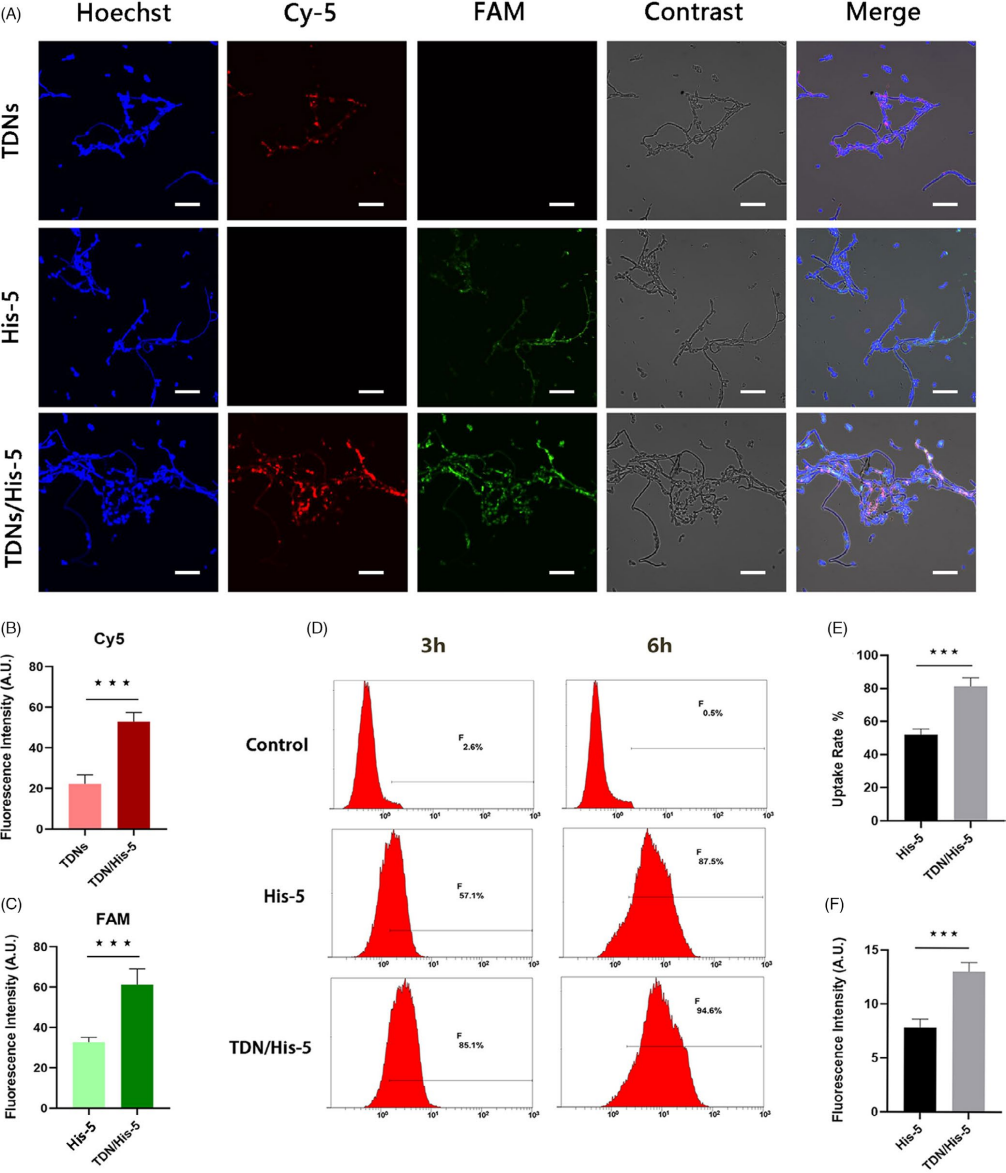
TDN/His‐5 complex uptake by *C. albicans*. (A) Confocal laser scanning microscopy images of *C. albicans* cells treated with 200 nm TDNs, 20 µm His‐5 and 200 nm TDN/His‐5 complex (1:100). Blue fluorescence and contrast images indicate the total number of live fungi. Red fluorescence and green fluorescence show the location of Cy5‐TDNs and FAM‐His‐5, respectively. Scale Bar: 50 µm (B) Analysis of the red fluorescence intensity of Cy5 in the confocal laser scanning microscopy images of the TDNs group and TDN/His‐5 group. (C) Analysis of the green fluorescence intensity of FAM in the confocal laser scanning microscopy images of the His‐5 group and TDN/His‐5 group. (D) Flow cytometry analysis of the uptake rates of 20 µm His‐5 and 200 nm TDN/His‐5 complex (1:100) by *C albicans* following incubation for 3 or 6 h. (E) Analysis of the uptake rate from the results of flow cytometry analysis after 3 h of incubation. (F) Analysis of the fluorescence intensity of the results of flow cytometry analysis after 6 h of incubation. Data are represented as means ± standard deviations, n = 3; statistical significance: *P <.05, **P <.01, ***P <.001

Immunofluorescence and flow cytometry were employed to evaluate the capability of *C. albicans* to take up TDNs, His‐5 and TDN/His‐5 complex. Figure 5A showed the confocal image of *C. albicans* incubated with 200 nm TDNs, 20 µm His‐5 and 200 nm TDN/His‐5 complex (1:100 mass ratio) for 2 hours. The fluorescence signals of Cy5 in TDNs group and FAM in His‐5 group showed that both His‐5 and TDNs can be internalized by cells individually. Moreover, the fluorescence signals of both Cy5 and FAM in the TDN/His‐5 complex group were stronger than those in TDN and His‐5 groups, indicating a greater uptake of TDNs and His‐5. Statistical analysis showed the obvious increase in fluorescence intensity of Cy5 and FAM in the TDN/His‐5 group compared with that in the TDNs and His‐5 groups (Figure 5B,C). Moreover, the spatial overlap between the red fluorescence emitted by Cy5 and green fluorescence emitted by FAM indicates the colocalization of TDNs and His‐5, revealing the stable synthesis of TDN/His‐5 complex.

The uptake efficiency of His‐5 was measured quantitatively between the His‐5 group and TDN/His‐5 group using flow cytometry. The uptake of His‐5 in the TDN/His‐5 complex group (81.2%, 95% CI: 69.4%–92.9%) was greater than that in the His‐5 group (52%, 95% CI: 44.1%–59.8%) after 3h of incubation (Figure 5D,E). After 6 hours of exposure, although there were no evident differences in the uptake rate between the two groups, the fluorescence intensity in the TDN/His‐5 group (13.0, 95% CI: 11.05‐14.94) was stronger than that in the His‐5 group (7.81, 95% CI: 6.05‐9.58) (Figure 5F), indicating that more His‐5 internalized in the TDN/His‐5 group.

In this study, the surface of His‐5 was modified by TDNs, thus allowing the anti‐fungal intracellular AMP His‐5 to be internalized by *C, albicans* and consequently promoting the pharmacological characteristics of His‐5. On the other hand, His‐5 can also enhance the transport efficiency of TDNs for *C. albicans*. Previous studies have proved that cationic penetrating peptide can assist DNA nanostructure like TDNs in cellular uptake.^29^, ^55^, ^56^ The immunofluorescence image indicated the similar result for fungi, which should be comprehensively researched in further studies.

The anti‐fungal efficacy of the TDN/His‐5 complex did not enhance gradually with the increasement in the proportion of TDNs in TDN/His‐5 complex. At the mass ratio of 50:1, the anti‐fungal effect of the complex decreased instead. Previous studies showed that TDNs can be internalized by cells and bacteria as the small‐weight‐drug vehicles.^23^, ^24^ We considered that the increased negative surface charges of the complex with more TDNs would weaken the electrostatic attraction between TDN/His‐5 and cell membrane, leading to decreased transport efficiency. Nevertheless, the above hypothesis needs to be confirmed by further studies to help us explore the transportation routes of TDN and His‐5 thoroughly.

From our perspective, surface modification of AMP by TDN may have broad applications, especially for fungi that are insensitive to traditional AMPs.

### 3.5 ROS formation and potassium efflux of *Candida albicans*

**FIGURE 6:**
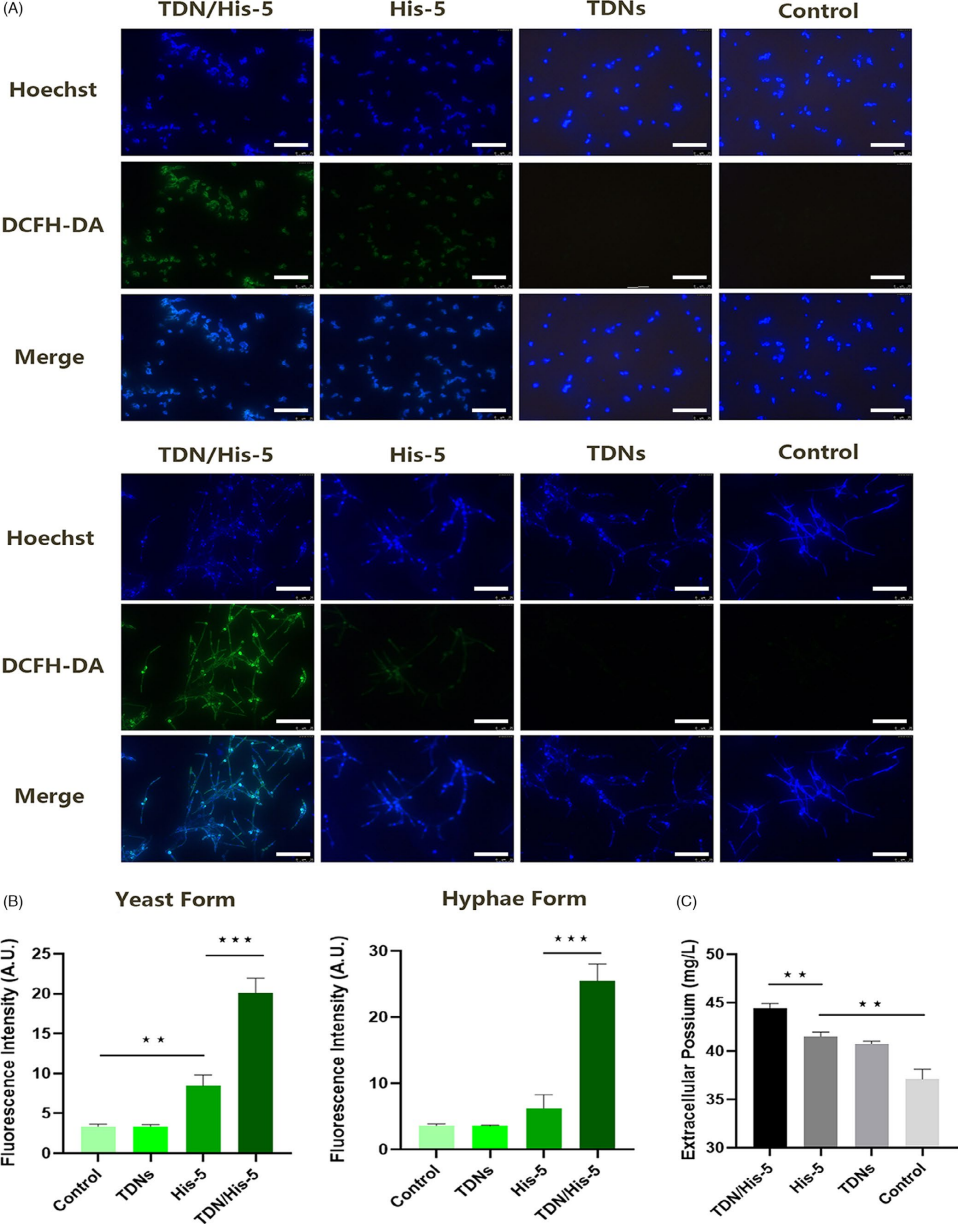
TDN/His‐5 induces the formation of ROS and potassium efflux in *C. albicans*. (A) Images of ROS formation in yeast and hyphal forms of *C. albicans* (200 nm TDNs, 20 µm His‐5 and 200 nm TDN/His‐5 complex [1:100] for 3 h). Blue fluorescence indicates the total number of *C. albicans*. Green fluorescence shows the formation of ROS in *C. albicans* cells. Scale Bar: 50µm (B) Analysis of the fluorescence intensity of ROS in confocal images. (C) Analysis of the concentration of extracellular potassium ions in *C. albicans* supernatant (200 nm TDNs, 20 µm His‐5 and 200 nm TDN/His‐5 complex [1:100] for 4 h). Data are represented as means ± standard deviations, n = 3; statistical difference: *P < .05, **P < .01, ***P < .001

To investigate the intracellular role of TDN/His‐5 complex, the early formation of ROS in both yeast and hyphal forms of *C. albicans* was monitored using the fluorescent probe DCFH‐DA.^57^, ^58^ As can be seen in Figure 6A,B, intracellular ROS production in the TDN/His‐5 group improved markedly compared with His‐5 group in both yeast and hyphal *C. albicans* cells. And the TDN group was not significantly different from the control group.^47^, ^57^, ^59^ The same results can be detected by flow cytometer, that the fluorescence intensity of DCF in the TDN/His‐5 group in yeast cells was much higher than other groups (Figure S2).

Potassium efflux caused by His‐5, which resulting in volume dysregulation and cell death, was also measured in this experiment (Figure 6C).^20^, ^32^, ^60^ The potassium concentration in the extracellular medium of the TDN/His‐5 group (44.45, 95% CI: 43.38‐45.51) significantly increased compared with that in the other groups. Moreover, the extracellular potassium levels in the TDN group (40.76, 95% CI: 40.16‐41.35) and His‐5 group (41.503, 95% CI: 40.47‐42.53) were significantly elevated compared with those in the control group.

### 3.6 Limitation

There are some limitations to our study. Only one pathogenic fungus was assessed, and detailed in vivo experiments were not conducted in this study, which we plan to perform in the future. In addition, the specific mechanism by which TDNs assist AMP in penetrating the fungal cell wall needs to be explored. We believe that the application of TDNs as the delivery vehicle of anti‐fungal AMP can be broadened by overcoming these limitations.

## CONFLICT OF INTERESTS

There are no conflicts to declare.

## AUTHOR CONTRIBUTIONS

Bowen Zhang involved in conceptualization, investigation, methodology, and project administration and wrote the original draft. Xin Qin involved in data curation, formal analysis and investigation and wrote the review and editing. Mi Zhou involved in methodology, investigation and software. Taoran Tian involved in data curation, methodology and resources. Yue Sun involved in data curation and investigation and wrote the review and editing. Songhang Li involved in data curation, methodology and software. Dexuan Xiao involved in resources and methodology. Xiaoxiao Cai involved in funding acquisition, supervision and wrote the review and editing.

## Supporting information

Figure S1‐S2

## Data Availability

The data that support the findings of this study are available from the corresponding author upon reasonable request.
