# Supplementary material for: Tetrahedral DNA nanostructure improves transport efficiency and anti‐fungal effect of histatin 5 against Candida albicans
Source: Cell Prolif. 2021 Mar 11;54(5):e13020. doi: 10.1111/cpr.13020 (PMC8088467; doi:10.1111/cpr.13020)
Supplement: Supplementary file 1 — Figure S1‐S2 [file CPR-54-e13020-s001.docx]

Supporting Information

**Tetrahedral DNA Nanostructure Improves Transport Efficiency and Antifungal Effect of Histatin 5 against *Candida albicans***

Bowen Zhang^1^†, Xin Qin^1^†, Mi Zhou^1^, Taoran Tian^1^, Yue Sun^1^, Songhang Li^1^, Dexuan Xiao^1^ and Xiaoxiao Cai^1^*

1 State Key Laboratory of Oral Diseases, National Clinical Research Center for Oral Diseases, West China Hospital of Stomatology, Sichuan University, Chengdu 610041, P.R. China

***Corresponding authors:**

Xiaoxiao Cai

E-mail address: [xcai@scu.edu.cn](mailto:xcai@scu.edu.cn)

^†^ Bowen Zhang and Xin Qin contributed equally to this work.


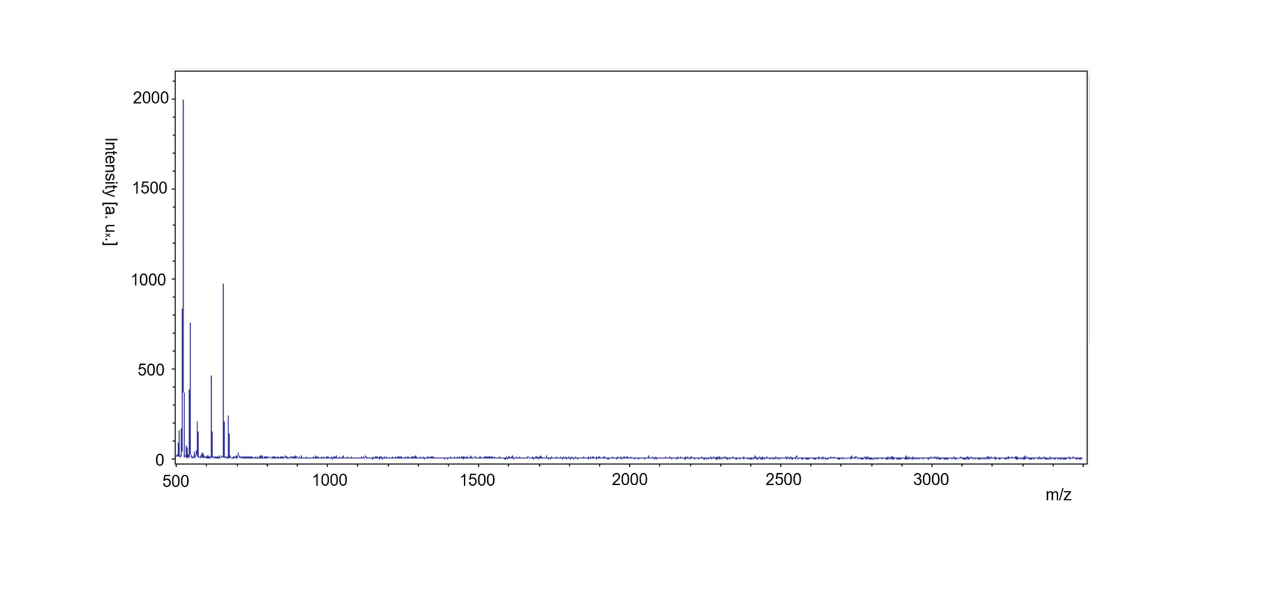


**Figure S1.** The MALDI-TOF MS profile of the 10% fetal calf serum medium. And it is clear that the profile of 10% fetal calf serum medium does not interfere with the profile of His-5 (2900-3000 *m/z*).


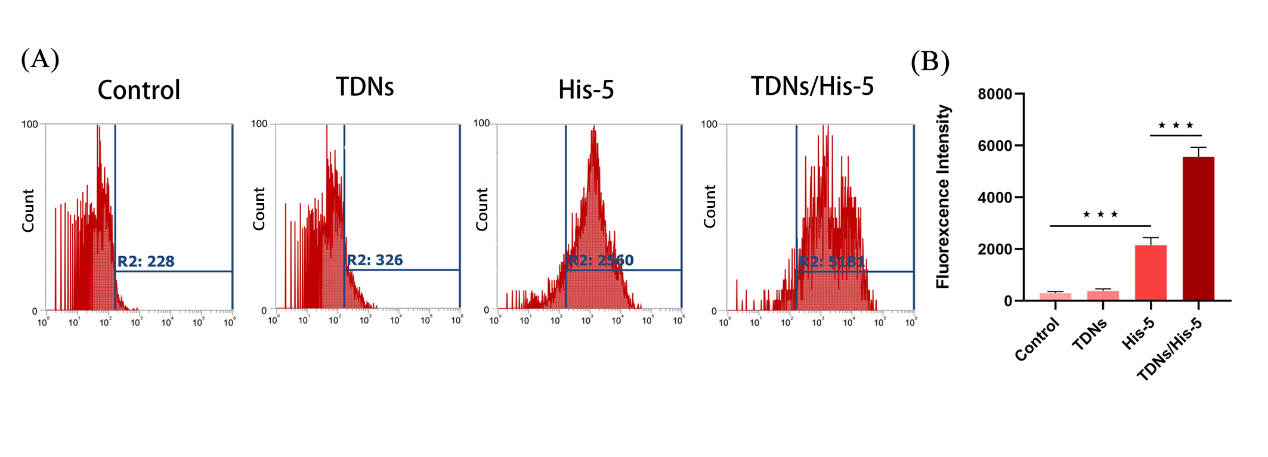


**Figure S2.** The formation of ROS in *C. albicans* detected by flow cytometry. (A) The profile of the fluorescence intensity of DCF in yeast cells detected by flow cytometry. (B) Analysis of the fluorescence intensity of DCF in yeast cells detected by flow cytometry. (5302, 95%CI: 4819-5891 for TDNs/His-5 group; 2231, 95%CI: 1911-2671 for His-5 group; 341, 95%CI: 231-431 for TDNs group; 228, 95%CI: 188-246 for control group) Data are represented as means ± standard deviations, n = 3; statistical difference: *p < 0.05, **p < 0.01, ***p < 0.001
